# A Critical Appraisal of Global Testing Protocols for Zoonotic Parasites in Imported Seafood Applied to Seafood Safety in Australia

**DOI:** 10.3390/foods9040448

**Published:** 2020-04-07

**Authors:** Michelle Williams, Marta Hernandez-Jover, Shokoofeh Shamsi

**Affiliations:** School of Animal and Veterinary Sciences & Graham Centre for Agricultural Innovation, Charles Sturt University, Wagga Wagga, NSW 2650, Australia; mhernandez-jover@csu.edu.au (M.H.-J.); sshamsi@csu.edu.au (S.S.)

**Keywords:** Australian seafood safety, international food safety, zoonotic parasites, Codex Alimentarius, imported seafood

## Abstract

It is not suggested that any country is intentionally exporting seafood which does not comply with Codex seafood-safety guidelines/codes/standards. However, with an open access resource such as fisheries, there is vast potential for errors to occur along convoluted supply chains, spanning multiple countries, which may negatively impact the safety of edible seafood products imported into Australia. Australian importation policy and inspection procedures are founded upon a bedrock of trust in the integrity, reliability and safety of the global seafood supply chain. In order for seafood imported to Australia to be considered safe the non-mandatory international health standards, governed by Codex Alimentarius, for seafood must be predicated upon the most efficacious methods and stringently governed by each exporting provenance. Currently, tests for zoonotic parasites are not applied to imported edible seafood products on arrival into Australia. Therefore, this critical analysis is aimed at discussing the effectiveness of current testing protocols for zoonotic parasites in edible seafood advised by Codex Alimentarius which may impact the safety of the product imported into Australia.

## 1. Introduction

Australia is one of the few countries globally which seems to have remained free of many zoonotic parasites and pathogens which are endemic to other countries [1]. Australian biosecurity is considered critical in the fight to reduce the risks posed by invasive pests and diseases [2] and as such is an essential national asset [3]. The introduction of white spot disease (WSD) into Australia is a propitious reminder of the severe economic and social consequences of a biosecurity breach. By 2016, the outbreak estimated cost to the Australian prawn industry was $49.5 million [4]. Despite fore-warnings from the global scientific community, of traded WSD infected frozen shrimp and an obvious spatial and temporal global spread [5,6,7], WSD was introduced into Australia. Whilst the introduction of WSD into Australia exposed a vulnerability in the nation’s biosecurity it also illustrated how difficult biosecurity defense becomes when exporters are prepared to flaunt international food safety recommendations. Australia has a great reliance on imported edible seafood products. No matter how highly regarded and efficient Australian biosecurity policy/procedures are, in a time or rapidly escalating global change, it is perhaps timely to re-evaluate the current international standards for zoonotic parasites in imported edible seafood in support of human health biosecurity in Australia.

Seafood is considered an important source of essential fatty acids, protein and B group vitamins [8] and is a valuable component of a healthy diet [9,10]. In Australia, the imported seafood product represents 70–75 percent of total Australian seafood consumption [11]. The Australian Bureau of Statistics [12] showed the cost of living rose 2 per cent in 2017, however, wage growth has been historically weak. Although Australian premium seafood products are available for purchase locally the cost may preclude the ‘average Australian’ from operating within this market. For example, locally caught flathead fillets retail for ~$54 per kg (October 2018) [13] whilst imported ‘South American Flathead’, a registered name in Australia for imported *Percophis brasiliensis* [14] retails for ~$20 less per kilo [15] and is an entirely different fish species. By implication it must be considered that the importation of seafood provides the Australian public with a stable, affordable and assessable source of dietary protein. Article 11 and 12 of the United Nations ‘*International Covenant on Economic, Social and Cultural Rights*’ [16] sets out in legal terms the basic human right to ‘food security’ which fulfils the concept of ‘nutrition’, ‘safety’ and ‘cultural legitimacy’. Articles 15–19 in the ‘*Voluntary guidelines to support the progressive realization of the right to adequate food in the context of national food security’* [17] detail ‘food security’ as a governmental responsibility under international law in the provision of ‘safe food’ as a basic human right. Article 15, stipulates the food must be ‘safe’ and article 16 stipulates it must be free of ‘unsafe substances’. In Australia, the Government has implemented policy and procedures to secure a food supply chain, internationally, which should provide ‘food security’ for all Australians. In Articles 17 and 19 of the ‘*Voluntary guidelines to support the progressive realization of the right to adequate food in the context of national food security’* [17] there seems to be clear application for the Government, should it be necessary, to update current inspection procedures for imported edible seafood to protect the basic human right of all Australians to ‘food security’. The aforementioned United Nations (UN) covenant [16] and the Food and Agricultural Organisation of the UN voluntary guidelines [17], appear to provide the Australian Government with autonomy to take any measures necessary to establish ‘food security’. However, in Article 3 of the World Trade Organisations ‘*General Agreement on Sanitary and Phytosanitary Measures*’ (*SPS*) [18], an international health standard becomes deductively legitimate if an exporting country can show compliance. Should an exporting country demonstrate compliance with an international health standard, even if this compliance represents less food safety than Australia as the importing nation desires, a downward adjustment in compatibility of Australian health standards may be necessary to facilitate trade. The international health standards referred to in the SPS are those set by Codex Alimentarius [18]. These international health standards are the global lynch pin for the safety of traded edible food and it would be expected these should be based on the latest scientific best practice. Therefore, the primary aim of this critical appraisal was to investigate if the current international standards for control of zoonotic parasites in seafood advised by Codex Alimentarius are adequate to ensure the safety of edible seafood imported into Australia. The secondary aim was to highlight cases of human illness from published literature where imported seafood was implicated as the agent of infection.

## 2. Methods Used for Critical Appraisal

**Figure 1:**
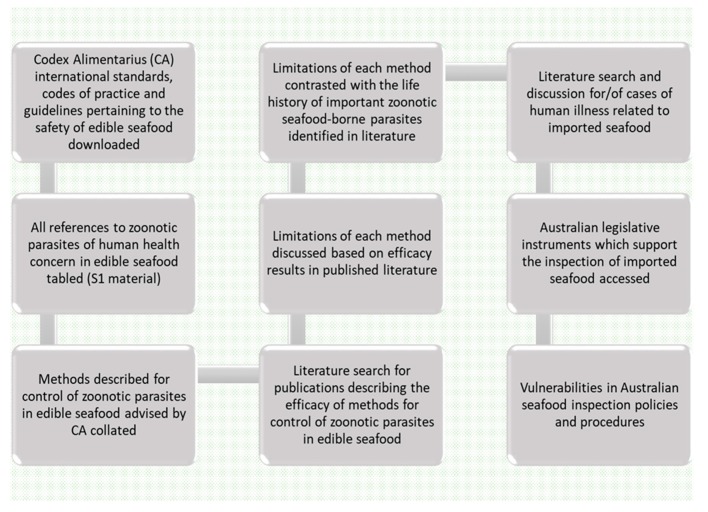
Flow diagram of the process used to develop the critical appraisal.

Please see Figure 1 for a flow chart of the steps followed to develop the critical appraisal. International food safety standards available at Codex Alimentarius were searched and all texts pertaining to seafood downloaded. All relevant Codex seafood safety advice pertaining to the control of seafood borne zoonotic parasites used for this critical appraisal is included in Appendix A. A literature search was conducted using Google Scholar and Charles Sturt University (CSU) Primo search engines for literature pertaining to the efficacy of each of the recommended control methods, candling, pepsin digestion, salting and brining for seafood borne zoonotic parasites advised by Codex. CSU Primo search engine automatically searches all major scientific journals such as Scopus and Web of Science which the university subscribes to. The same search engines were used to access literature which described the life history of seafood borne zoonotic parasites nematodes, cestodes, myxozoa and trematodes to contrast against each control method advised by Codex. Finally, the current Australian legislative instruments which support the inspection of seafood imported to Australia were accessed and critically analysed for weaknesses based on all of the above.

## 3. Codex Alimentarius Non-Mandatory Recommendations

**Figure 2:**
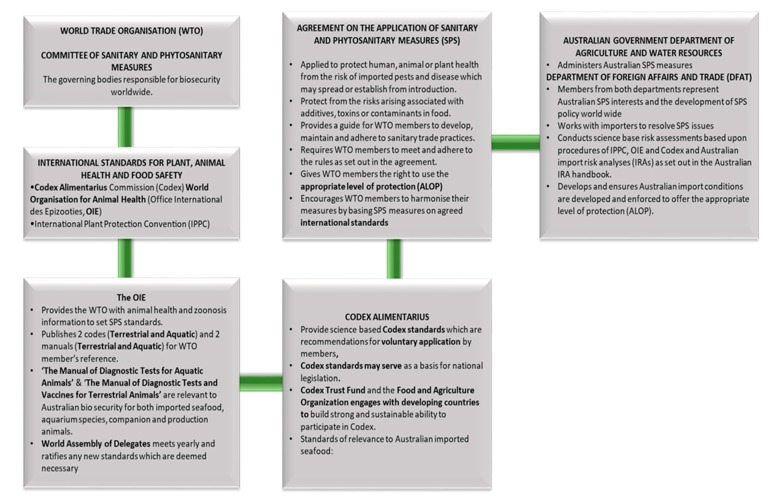
The global processes which relate to the safety of edible seafood on arrival into Australia. Codex food safety standards are not mandatory or enforced however it is expected that member nations, such as Australia, of the World Trade Organisation will be responsible in implementing the advised international food safety standards. Australia has a great reliance on the testing procedures of the exporting country to ensure imported seafood products are free of parasites and safe for human consumption. Original figure developed from information at [18].

Please see Figure 2 for how the non-mandatory Codex international food safety guidelines/codes of practice/standards are developed.

### 3.1. Candling

Codex Alimentarius ‘Code of Practice for Fish and Fishery Products’ defines candling for parasites as “passing fillets of fish over a translucent table illuminated from below to detect parasites and other defects” [19]. Section 8.1.6 of the code provides technical advice for candling and recognises “viable parasites” in fish as a potential biological hazard (p. 103). The candling line is recommended to be “continuous and sequential to permit uniform flow without stoppages or slowdowns and removal of waste” (Step 1, p. 103). Codex does not recommend candling in conjunction with pressing in any of the codes or standards which contain a reference to parasites. ‘Pressing’ involves placing the sample of fish between two thin acrylic sheets and examining under an appropriate light source [20]. Table A1 in Appendix A shows Codex recommendations for candling to control parasites for specific seafood products [19] ‘Code of Practice for Fish and Fishery Products’ still apply. Codex Alimentarius is the global food safety authority and therefore the advised methods for parasite detection should unquestionably be the most effective available.

### 3.2. Operator Constraints and Candling Accuracy

According to Andreoletti, et al. [21] an experienced ‘Candler’ can examine up to 300 fillets an hour. In order for an operator to examine 300 fillets per hour, every 12 s a fillet must be checked and parasites removed. If CODEX STAN 165-1989; CODEX STAN 190-1995 and CODEX STAN 311-2013 (See Table A1, Appendix A) [22,23,24] apply then operators along the candling line must additionally decide if there are more than 2 parasites per kilo, if the parasite capsule is >3 mm and if the un-encapsulated parasite is >10 mm. A questionnaire distributed to fish processors in Scotland highlighted that few carried out any comprehensive examination of fish for larval nematodes. Only one processor used a candling table and commented that the candling method was limited in thick skin or fleshed fish and another said it was not cost effective [25]. Visual fatigue after prolonged periods of observation has been demonstrated to affect diagnostic accuracy [26]. Wootten and Cann [27] comment that operator eye fatigue is rapid and during extended periods of candling the efficiency of the method may be impaired. It cannot be discounted that operator fatigue may limit the efficacy of parasite identification along the candling line. Candling has been demonstrated to be 15% less efficacious under commercial working conditions [28] which may support observer fatigue as significant within parasite identification and removal.

### 3.3. Limitations of Candling to Detect Parasites

#### 3.3.1. Nematodes

It has been noted that the efficiency of candling as a technique has limitations due to low penetration into fish muscle of the white light used [28]. As a method is also considered ineffective in bright light [21]. McGladdery [29] considers the technique effective for detection of *Pseudoterranova* spp., which are darker, but limited in detecting smaller, white worms, such as *Anisakis* spp. However, only 31.7% (143/450) *Pseudoterranova* larvae were identified in monkfish fillets using white light candling [25] which is in contradiction of McGladdery [29]. Candling combined with pressing has been demonstrated to be more efficacious to detect parasites in fish than candling alone [20]. There is a great variability between each inspection method in terms of ‘hours of labour’ which are required. As a result, white light candling may be based upon convenience rather than safety best practice. According to Codex [19] candling carried out by skilled personal in a suitable location is effective in the control of parasites when implicated species of fish are used (step 3, p. 104). However, in Annex 1 “potential hazards associated with fresh fish, shellfish and other aquatic invertebrates”, Section 1.1 it is considered that “candling, trimming belly flaps and physically removing the parasite cysts will also reduce the hazards but may not eliminate them” [19]. The effectivity of candling in the same Codex code of practice [19] has been described as both effective and ineffective in controlling seafood-borne parasites. Inconsistencies also appear in recommendations CAC/RCP 1-1969: “No raw material or ingredient should be accepted by an establishment if it is known to contain parasites”, [30] and CAC/RCP 52-2003 [19] “Unless they can be reduced to an acceptable level by normal sorting and/or processing, no fish, shellfish or other aquatic invertebrates should be accepted if they are known to contain parasites”. If candling does not completely eliminate the parasite hazard and seafood should not be accepted if it contains parasites then reducing the parasites to an acceptable level appears contradictory. Levsen et al. [31], in a study of fish from the Northeast Atlantic Norwegian spring spawning (NSS) herring (*Clupea harengus*), blue whiting (*Micromesistius poutassou*), and mackerel (*Scomber scombrus*) demonstrated only 7 to 10 percent of the nematode *Anisakis* larvae present in the fillets of all fish species were detected by candling. In NSS herring and blue whiting the detection efficiency of candling was decreased as fillet thickness increased. In blue whiting, the detection efficiency of candling with UV light was only 10–15% despite the average fillet thickness of 11 mm. Adams, et al. [32] in contrast had relatively high recovery of *Anisakis* larvae from four types of white fleshed fish; rockfish (*Sebastes* spp.), arrowtooth flounder (*Atheresthas stomias*), sole spp. (family Pleuronectidae) and true cod (*Gadus macrocephalus*); utilising the candling method identified 43% to 76% of the anisakids present. However as one viable L3 larvae can result in human infection the method does not completely eliminate the danger. In fillets of monkfish (*Lophius piscatorius*) and cod (*Gadus morhua*) candling was only successful in identifying 16.8% and 33.3% of *Anisakis* and 31.8% and 53.6% of *Pseudoterranova* respectively which were present [25]. A time saving method to identify parasites in fish is recommended which candles a representative sample of fillets from a batch [27], however, this assumes that parasitism is equal between fish of the same species from the same location. The number of *A. simplex* larvae in mackerel and blue whiting fillets was from 0–19 and 0–71 respectively in fish caught in the same location [31].

#### 3.3.2. Trematode Metacercariae

The conventional method for detection of zoonotic trematode metacercariae in fish include microscopic examination of compressed flesh samples which according to Andreoletti et al. [21] is time consuming and lacks sensitivity. However, Murrell and Sohn [33] concluded that this method was economical, time effective and determined the exact location of the metacercariae. Andreoletti et al. [21] comment that individual fish harbour few metacercariae so it is difficult to estimate infection intensities. A total of 113 freshwater fish species, mostly cyprinids, have been recorded as hosts for metacercariae of zoonotic flukes [34]. Species of cyprinid fish commercially available in Laos were identified infected with zoonotic metacercariae (number of fish species infected: intensity range), *Opisthorchis viverrini*, 6: 1–6980; and *Haplorchis yokogawai*, 3: 1–1370 [35]. Commercially available fish from a Chinese market were identified infected with zoonotic metacercariae of *Haplorchis taichui*, 10: 1–485; *Haplorchis pumilio*, 10: 3–312; *Centrocestus formosanus*, 5: 1–32 and *Metagonimus yokogawai*, 11: 1–1836 [36]. The recovery rate of zoonotic metacercariae in tilapia and catfish fillets using the candling method according to Murrell and Sohn [33] is 53% and 68% respectively. Metacercaria in fish range in size; Opisthorchiidae 0.1–0.15 mm; Heteropyhidae and Echinostomatidae 0.14–0.16 mm [33] and in cases of intense infection it may be impossible to remove all infectious metacercariae and discarding the fish the only option. In recent times, and in accordance with Article 9 of the SPS [18], critical control intervention programs have been implemented in some Vietnamese aquaculture facilities. These programs have had some success in lowering the burden of infection metacercariae in cultured fish [33] and are a promising initiative. Although not recommended by Codex Alimentarius [37] the Vietnamese catfish industry, mainly driven by the implementation of western quality standards [38] has also taken the initiative to use the press method of candling for metacercariae.

#### 3.3.3. Tapeworm Plerocercoids

Rozas et al. [39] comments that the press method in conjunction with candling provides more effective detection of plerocercoids in fish muscle than candling alone. Torres and Puga [40] compared three methods of candling to isolate plerocercoids (N = 310) in trout fillets. The candling method as advised by Codex had a 22% detection efficacy. A combined slice and candle had 40.8% and press method combined with candling had 59.2% efficacy. When all of the three candling methods were combined there was 90.9% detection efficacy however the total procedures included sectioning muscle tissue and examination of up to 18 compression plates. The incidence and mean intensity of infection of *Diphyllobothrium dendriticum* (syn. *Dibothriocephalus dendriticus*) plerocercoids in fish has been identified at 83.2% and 8.8% [41] and *D. latum* (syn. *D. latus*) the mean infection intensity has been identified as low as 1.25 parasites/fish [42].

#### 3.3.4. Myxozoans

Species of seafood-borne zoonotic myxozoa are not included as a human health concern in any of Codex seafood safety guidelines. There is one mention of myxosporidia which may hinder the production of surimi due to myoliquefaction of fish muscle. The same source provides a recommendation of the best method to successfully bind infected fish muscle into surimi for human consumption [43]. Olive flounder from Japanese waters have been identified infected with three myxosporean species; *Kudoa septempunctata*, *K. thyrsites*, and *K. shiomitsui* [44]. Imported farmed olive flounder have been demonstrated infected with *K. septempunctata* [45]. Yellowfin, Bigeye and Bluefin tuna have been identified infected with the zoonotic *K. neothunni* [46,47] and *K. hexapunctata* has been identified in Bluefin [48] and Yellowfin tuna [46]. It should be noted that in samples of Northern Bluefin tuna (*Thunnus thynnus*), obtained from nine different countries, only the samples of Japanese origin were identified infected with *K. hexapunctata*. Species *K. neothunni* and *K. septempunctata* do not form a cyst or pseudocyst, [45,47] and even if inspected for parasites it is doubtful these parasite species would be detected macroscopically.

### 3.4. Ambiguity in Codex Food Safety Guidelines

CODEX STAN 165-1989; CODEX STAN 190-1995 and CODEX STAN 311-2013 [22,23,24] state there should not be “two or more parasites per kg of the sample unit detected by candling”. The three standards apply to frozen and smoked products and as freezing is determined by Codex to eliminate the zoonotic potential of all parasites this inclusion seems irrelevant. There are no other Codex standards which clarify what an ‘acceptable’ number of parasites per kg may be despite “reduced to an acceptable level” being used in CAC/RCP 1-1969 [30] and CAC/RCP 52-2003 [19] in regard to seafood-borne parasites. In CAC/GL 88-2016 [49] there are six separate references to “acceptable” limits of parasites in fish without any clarification of what an acceptable number may be. CODEX STAN 311-2013 [24] states that viability of, and killing method for parasites may be determined using methods “acceptable to the competent authority having jurisdiction”. The guidelines use of “acceptable” may allow a subjective interpretation of what is in essence an unmeasurable amount and an interpretative administration of seafood safety standards which may vary significantly between processors and regions. In Codex standard 244-2004, “Standard for Salted Atlantic Herring and Salted Sprat” [50] Annex III, Point 2, states irrespective of the presence of visible parasites which may be seen in the sample unit (Annex III, Point 1), ‘the verification of the presence of parasites in intermediate entire fishery products in bulk intended for further processing could be carried out at a later stage” (p. 8). It is unclear when the later stage may be and ‘later stage’ seems to be an indefinite term particularly when applied to fish species demonstrated to have high intensity of infection with Anisakids. Baltic herring have a demonstrated infection intensity of 20–50 in larger fish. Further, intensity of infection has shown a rapid 30–40% increase in a five-year period [51].

### 3.5. Ambiguity of Codex Salting and Brining Recommendations

In CODEX STAN 244-2004 [50] 3.1 “Fish flesh shall not be obviously infested by parasites” and “If living nematodes are confirmed, products must not be placed on the market for human consumption before they are treated in conformity with the methods laid down in Annex II”. In the most recent version accessed (2018) Annex II states **“**the adequate combination of salt content and storage time (to be elaborated)—or by other processes with the equivalent effect (to be elaborated).” In CAC/RCP 52-2003 [19] 12.1 “Where appropriate, fresh fish intended for processing salted fish should be checked for visible parasites” and “an adequate combination of salt content and storage time can be used as treatment procedures for killing living parasites”. It, again, is unclear where ‘appropriate’ may be along the food chain or if processors would consider salted fish in need of checking for parasites. Adequate salt concentration or storage time required to kill parasites is not defined at all. In the same standard, Section 2.2.2. includes the categories for salted fish and the percentage of salt required in the muscle of the fish during the water phase. These include 2.2.2.1 very lightly salted: >1% salt and ≤4%; 2.2.2.2 lightly salted: >4% and ≤10%; 2.2.2.3 medium salted: >10% and ≤20%; 2.2.2.4 heavily salted: >20%. Herring at 15–19% brine were found infected with a number of live *Anisakis* larvae and 22%–23% brine was required to kill nematode larvae over a period of 7 days which commenced 3–4 days post salting [52]. According to Lubieniecki [52], the salt concentration of herring flesh was influenced by brine salt concentration, but also additionally the gonad maturity stage, lipid content of the flesh, and salting temperature and hence are factors which may contribute to increase the viability of *Anisakis* larvae. Three subsequent studies conducted by Grabda (1971–1973) confirmed that live *Anisakis* larvae were able to survive in 15–19% brined herring [as cited by 53]. In a study of fresh Baltic herring, after a week at a 5.6% visceral salinity, 98.2% of *Anisakis* larvae in herring were motile; 2 weeks at salinity of 9.36%–12.9% no motile larvae were observed however after culture 25/25 of the non-motile larvae became motile again over a three-day period. At three weeks visceral salinity, 11.6%–14.04%, 13/71 larvae identified became motile on Day Two of culture. After four weeks at 12.2%–14.6% salinity no motile larvae were found in the cultures [53]. It appears that under Codex definitions of ‘very lightly’, ‘lightly’ and ‘medium’ salted that it would be after four weeks from the initial date salting commenced that the fish product could be regarded as entirely safe for human consumption. Oh, et al. [54] demonstrated *Anisakis* larvae were viable after seven days emersion in 5% NaCl (81.7%) and 10% NaCl (26.7%). All larvae were inactivated after seven days in 15% NaCl, and six days in 20% NaCl. Most larvae survived in all NaCl concentrations for 3–12 h. However, in this study larvae were introduced directly into brine. It is possible that larvae in fish musculature, where saline penetrates more slowly, may demonstrate longer inactivation times. The slow inactivation of infectious larvae and the regeneration of moribund larvae presumed dead is concerning. This implies that larvae in salted products may become infectious after consumption. Codex stipulates in CODEX STAN 244-2004 and CODEX STAN 311-2013 [24,50] that a viable larvae is one which clearly demonstrates spontaneous movement after mechanical stimulation. By implication the moribund larvae, in the studies cited, which became viable after incubation would be considered non-viable according to Codex recommendations. In the case of *Diphyllobothrium* spp. (syn. *Dibothriocephalus*) in fish, salting in 10% to 20% NaCl solution has been demonstrated to kill the plerocercoids after 1 or 2 h [55]. Freshwater fish *Pseudorasbora parva* infected with metacercariae of *Clonorchis sinensis* were treated with a heavy salt (fish/salt = 10 gm/3 gm) and kept at 26 °C for 5–15 days. Metacercariae remained viable and produced infection in rats up to seven days after salting [56].

## 4. Human Health Risks Posed by Seafood-Borne Parasites

There are many seafood-borne zoonotic parasites which have been implicated in cases of human infection. For a comprehensive list of seafood-borne zoonotic parasites which may be a human health concern in imported edible seafood please see Shamsi and Sheorey [57]. Cooking and freezing according to the methods described in the relevant Codex standards is sufficient to kill all zoonotic parasites in seafood. At present the nematode *A. simplex* is the only species known to cause allergic reactions of varying exigency [58], with killed parasites in fish representing an allergen risk to some [59]. The consumption of raw or improperly cooked seafood is an important risk factor for humans acquiring a seafood-borne parasite zoonosis [32].

The World Health Organisation and the Food and Agricultural Organisation (FAO) in a review of parasites within the food trade concluded that the complex life cycle of aquatic parasites allows great potential for contamination of edible seafood. Further, the panel commented that food-borne parasitic diseases were neglected and underreported globally [60]. The WHO Foodborne Disease Epidemiology Reference Group [61] observed that the full human health impact of parasites in food is unknown. During the joint WHO/FAO review 6/24 parasites evaluated pertained to those in edible seafood products [60]; Anisakidae rated four according to trade risk. Sumner and Ross [62] in a 2000 Australian risk assessment awarded a low hazard for ‘parasites in sushi/sashimi’; the only pairing relating to parasites in seafood. In 2012, an Australian risk assessment of zoonotic parasites in Australian fish [63] commented that freshwater fish are not used for sushi/sashimi, however, identified anisakidosis/anisakiasis as underreported and/or misdiagnosed within Australia. In a 2015 risk assessment of Australian fish used for sashimi the authors concluded that the low incidence of anisakidosis in Australia may be due to underreporting or elimination of the parasite hazard during processing and preparation [64]. However imported fish and the potential for fish substitution was not included in this risk assessment or in a subsequent Australian risk assessment in 2017 [65]. Warner, et al. [66] reported that 58% of fish samples obtained from sushi venues in the Miami/Fort Lauderdale-area were mislabelled and 100% of Snapper was incorrectly labelled. It is unclear if mislabelling seafood is a significant risk in Australia.

Opisthorchiasis and clonorchiasis have been increasingly reported from non-endemic areas [67]. An outbreak of acute opisthorchiasis in an Israeli family was reported after eating illegally imported raw carp [68]. Opisthorchiasis has also been reported in native Hawaiians after consuming imported fish from endemic areas of infection [67]. Rohela et al. [69] regarding *Clonorchis sinensis*, and Park [70] regarding *Opisthorchis viverrini* and *Clonorchis sinensis*, commented that human infection outside traditional areas occurs as a result of consuming frozen, dried or pickled imported freshwater fish infected with metacercariae. Human infection in Hawaii with *Clonorchis sinensis* has been attributed to the consumption of dried or pickled fish imported from endemic areas [71]. Infected salted, dried or pickled fish is a significant risk factor in the transmission dynamics of *Opisthorchis viverrini* [72]. The importation of intensively farmed native fish, cyprinid carp species [60], Catfish/Basa, (*Pangasianodon hypothalamus*) and Tilapia, (*Orechromis niloticus/O. mossambicus*) which are susceptible to infection, has great potentiality to cause zoonotic infection in geographic regions outside the normal areas of endemicity [60,73]. Evidence in Vietnamese and Chinese aquaculture according to Murrell et al. [73] would suggest the potential contamination with zoonotic seafood-borne trematodes of seafood destined for international trade. The authors further advise that seafood imported from areas of parasite endemicity, particularly Asia, may be an infection risk to consumers and prevention should be implemented throughout the market chain. The global fish trade is considered an important factor in the alteration of the traditional geographical boundaries associated with *Diphyllobothrium* spp. (syn. *Dibothriocephalus*) [74]. The consumption of imported fish has been linked to cases of human infection in Spain [74,75,76], France [77,78], Switzerland [79,80,81] and recently the first two cases in Singapore from *D. nihonkaiensis* [82] which may indicate a deficit in the inspection processes of the exporting countries. Ogata, et al. [83] considers imported/introduced tilapia, farmed in Mexico since 1964, for the increase in gnathostomiasis cases regionally. In America, live eels imported for human consumption from Asia [84] and Vietnam [85] have been demonstrated heavily infected with encysted and un-encysted *Gnathostoma* spp. larvae. Imported fish has been implicated in cases of human infection from *Capillaria philippinensis* in Egypt (Youssef et al., 1989), which has been hypothesized as the entry point of the parasite into Egypt [86]. Infestation of fish for human consumption by anisakid nematodes has increased markedly during the last 20 years [87]. Cooking or freezing does not destroy the allergenic capacity of *A. simplex* which has been implicated in human reactions to canned fish [88]. *A. simplex* allergens have been identified in baby food products containing plaice and European hake [87]. No mention is made of the allergenic potential of *A. simplex* in canned products in any Codex recommendations. Mossali et al. [87] considers the frequent presence of anisakids in processed food reflects a utilisation of poor quality fish which would normally be discarded. The European Food Safety Authority (EFSA) has introduced a requirement for the routine testing of canned fish for anisakids using PCR method [21,89]. Australia imports a significant quantity of canned fish from many European countries [90] and these include species of fish high risk for human anisakiasis [58,91,92,93,94]. It is unknown if any of these canned products pose an Australian human health biosecurity risk.

## 5. Imported Seafood Inspection in Australia

**Figure 3:**
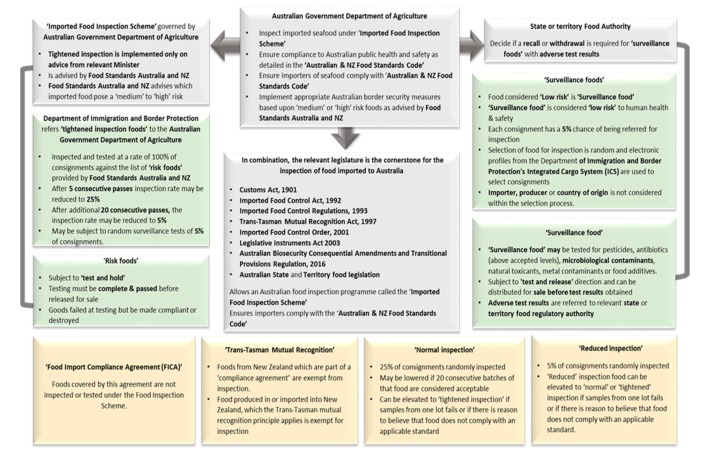
Representation of the legislative instruments that support the inspection of seafood imported to Australia in grey, the testing regimes for ‘surveillance’ and ‘tightened’ inspection for ‘risk foods’ in green. In yellow is the testing regime for ‘normal’, ‘reduced’, ‘compliance’ and foods which have been classified under the ‘Trans-Tasman Mutual Recognition Agreement’. ‘Compliance agreements’ are entered into voluntarily by the exporting provenance which must show compliance and equivalency with the standard of their food management systems which is audited annually. The original figure was developed from information at [18,95,96].

**Figure 4:**
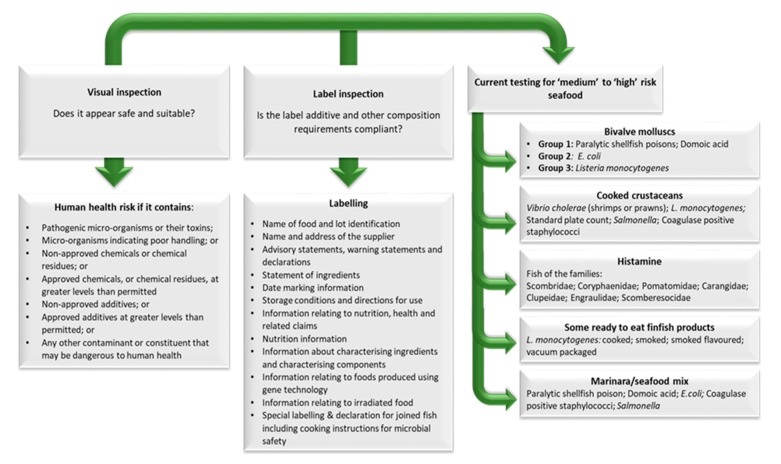
Representation of the current tests applied to ‘risk’ food on entry to Australia. All ‘risk’ food is inspected at the rate included in Figure 2. ‘Risk’ food will be examined visually and the label checked. The tests applied at present to five groups of ‘risk’ foods have been included. Currently no additional tests are applied to ‘risk’ foods for detection of zoonotic parasites in imported edible seafood. Visual inspection may be effective to identify some macroscopic parasites in seafood however as a tool it is extremely limited as most parasites infecting seafood require microscopic inspection by a trained professional. Original figure developed from information at [18,95,96,97,98,99].

At present, there are no additional tests applied to imported edible seafood, for detection of zoonotic parasites, on entry to Australia. Figure 3 and Figure 4 describe current tests applied to imported edible seafood on entry to Australian.

As related in an email from an Australian Government Biosecurity Officer on the 26th August, 2019 “lesions on fish caused by parasites are not considered to be either a biosecurity (regulated by my section) or human health (regulated by the Imported Food Inspection Scheme) risk”. Of the 29 Schedules in the ‘*Australian and New Zealand Food Standards Code*’ [97] there are none which relate to zoonotic parasite contamination of imported edible seafood. Edible seafood may be subject to label inspection as detailed in the Part 1 and Part 2.2 (2.2.3 Fish and Fish Products) of the ANZFSC [97] and visual inspection as detailed in Sections 3(a)(vii) and 3(b) *Imported Food Control Act 1992* [95]. Visual inspection is based upon Section 3(a)(vii) “any other contaminant or constituent that may be dangerous to human health” and 3(b) “it has been manufactured or transported under conditions which render it dangerous or unfit for human consumption”. Certainly, visual inspection could be interpreted to include visual detection of zoonotic parasites. However, as a tool this is inadequate to detect parasite contamination [100]. For example, in a 2007 study only 26/185 *Anisakis* larvae in monkfish fillets [25] were identified using visual inspection. Microscopic examination, candling [32], UV light [31], PCR [89] and pepsin digestion method [101] by a trained professional are all valid methods but are not listed as the tests applied to either ‘risk’ or ‘surveillance food’ within the Australian food inspection scheme. There have been six import consignments failed in the time period 2010–2018 based upon visual inspection [102]. None of the fails were as a result of parasites visualised in edible seafood. *The Imported Food Control Amendment Bill 2017* [103] was passed by the Australian House of Representatives on 11/9/2018 [104] and amends the *Imported Food Control Act 1992*. The amendments have been designed to place the onus of responsibility on the exporters to provide documentary evidence of their adherence to internationally recognised food safety controls. An impact statement was circulated by the Government during August 2016 pursuant to the Imported Food Control Amendment Bill 2017 [105]. There was no mention of parasites related to seafood in the impact statement [106]. Under Part 4 (35A [1,2,3,4,5,6,7,8,9,10]) of the *Imported Food Control Act 1992* [95] food exporters may voluntarily enter an agreement with Department of Agriculture for a ‘Food Import Compliance Agreement.’ Imported food under this agreement is not inspected or tested under the Food Inspection Scheme [98]. The exporters documented food management system must comply with Australian and New Zealand Food Standards Code [97] and Australian Standard ISO 22000:2005 *(Food safety management systems-requirements for any organization in the food chain)* [107]. Australian inspection processes for imported seafood places significant trust in the exporting nations ‘equivalency’ in testing procedures and adherence to the Sanitary and Phytosanitary Measures Agreement, 1995 [18].

## 6. Discussion

During 2017, Australia imported a significant amount of seafood from countries endemic for infection with zoonotic parasites. At the time of writing (15/9/2019) *The Australia New Zealand Food Standards Code* [108], Standard 3.2.1—‘Food Safety Programs’, Standard 3.2.2—‘Food Safety Practices and General Requirements’ make no mention of parasite risk in local or imported fish or standards if fish is to be consumed raw. Standard 4.2.1 ‘Primary production and processing standard for seafood’ (Australia Only) list parasites as a possible contaminant however Standard 1.6.1, table to section S27—4, of ANZFSC which should list the maximum allowable levels of contamination does not contain information for parasites at all [108]. The *‘Export Control (Fish and Fish Products) Orders 2005*′ [109] which guides our export policy makes no mention of parasites. The Food Safety Information Council of Australia comments that there is a slight risk associated with the consumption of raw seafood, sushi and sashimi for example, but these risks can be mitigated by consuming seafood from safe waters, chilling and correctly storing or purchasing from licensed suppliers. No mention is made of parasites in raw fish or freezing before consuming raw [110], however, in a 2005 ‘Safe Seafood Australia’ publication there is a recommendation to freeze fish (Australia only) if intended for raw consumption [111]. ‘*The Compendium of Microbiological Criteria for Food (2018)’ from Food Standards Australia and New Zealand* in Appendix I mentions parasites as a possible pathogenic microorganism which can cause foodborne illness however this is mentioned only once in the document [112]. Safefish is funded by the FRDC and is concerned with Australian seafood safety and trade. The ‘seafood safety fact sheets’ (2015) produced by Safefish make no mention of parasites in seafood [113]. At present, there is a paucity of information regarding zoonotic seafood borne parasites in Australia and it is not surprising that seafood borne parasitic disease is almost unknown. Globally, diseases from food-borne parasites are often neglected by Governmental health authorities and official figures are not reflective of the prevalence or incidence of disease [60]. The WHO Foodborne Disease Epidemiology Reference Group [61] commented that despite food borne diseases being a significant worldwide cause of death and morbidity the full impact of parasites in food is unknown. According to Kirk, et al. [114] within Australia only 28% of people affected with food-borne illness will seek medical attention. Absence of reported cases of seafood-borne parasitic disease have been used as evidence that there is no disease in Australia [115]. However, according to Shamsi and Sheorey [57] misdiagnosis in Australia contributes to lack of evidence regarding the prevalence of seafood-borne zoonoses and reliable parasite focused epidemiological data [116]. In Australia, where zoonotic parasites are largely unrecognised [57], the lack of reported cases of seafood-borne parasitic disease may more clearly reflect lack of diagnostic suspicion rather than absence of disease.

## 7. Conclusions

It is unlikely that Australia is immune from seafood-borne parasitic disease which has been widely recognised internationally. The intense cultivation of aquaculture species and the international trade in both farmed and wild caught seafood is a key factor in establishing global food security. However, these same endeavors which bring seafood to all corners of the globe are also high risk for the spread of pathogens and zoonotic parasites. As a member nation of the World Trade Organisation Australia is bound to uphold the three agreements signed at the Uruguay round of talks collectively known as the ‘Marrakesh Agreement’ [99,117,118] and designed to facilitate between country trade. Articles 3, 5 and Article 10 of the SPS encumbers Australian policy makers from implementing any additional testing procedures for imported edible seafood which would hinder between country trade without robust scientific justification. Support of developing nations to reach international safety and health standards is a requirement in Article 9 of the SPS [18]. Rather than a downregulation of health standards to comply with international trade agreements perhaps increased support of the seafood industry in developing nations to achieve upregulation of food safety compliance may be a positive step forward. Australian biosecurity is considered exemplary. However, as the onus for inspection of seafood imported to Australia is increasingly awarded to exporters the issue is focused away from the strength of Australian food biosecurity towards trust in the international food safety standards.

## Appendix A

**Table A1:** Appendix A Represents all Codex Alimentarius food safety codes of practice/standards and guidelines relating to zoonotic parasites in seafood. In orange are those documents which contain specific advice pertaining to zoonotic parasite and those which do not are shaded in green. Table developed from information at Food and Agriculture Organisation (2018) and information is taken verbatim from each of the codes of practice/standards and guidelines which relate to control of zoonotic parasites.

Codex Codes of Practice	Codex Code of Practice which Relate to Parasite in Fish Processing
**CAC/RCP 1-1969** (Last updated 2003) General principles of food hygiene	**5.3 INCOMING MATERIAL REQUIREMENTS** No raw material or ingredient should be accepted by an establishment if it is known to contain **parasites**, undesirable micro-organisms, pesticides, veterinary drugs or toxic, decomposed or extraneous substances which would not be reduced to an acceptable level by normal sorting and/or processing. Where appropriate, specifications for raw materials should be identified and applied.
**CAC/RCP 52-2003** (Last updated 2016) Code of practice for fish and fishery products	**2.5 Candling** Passing fillets of fish over a translucent Filluminated from below to detect parasites and other defects. **Hot** is generally sufficient to kill parasites, to destroy non-sporulated bacterial pathogens and to injure spores of human health concern.
	**5**. Unless they can be reduced to an acceptable level by normal sorting and/or processing, no fish, shellfish or other aquatic invertebrates should be accepted if they are known to contain **parasites. 5.2 Parasites of public health significance:** trematodes, nematodes, cestodes
	**6.2** Infection with **nematode parasites** is absent from, or very much reduced in, farmed salmon compared with salmon caught in the wild
	**9.1.1. Raw, fresh or frozen fish reception** Potential hazards: microbiological contamination, viable parasites
	Training in species identification and communication in product specification should be provided to fish handlers and appropriate personnel to ensure a safe source of incoming fish where written protocols exist. Warranting special consideration are the reception and sorting of fish species that pose a risk from **parasites. 9.1.3. Frozen storage** Potential hazards: microbiological contamination, toxins, viable **parasites**. For killing **parasites** harmful to human health, the freezing temperature and monitoring of duration of freezing should be combined with good inventory control to ensure sufficient cold treatment. **9.1.6 Filleting, skinning, trimming and candling** Potential hazards: viable parasites, Potential defects: **parasites. 9.1.6. Candling of skinless fillets** by skilled personnel, in a suitable location that optimizes the illuminating effect, is an effective technique in controlling **parasites** (in fresh fish) and should be employed when implicated fish species are being used.
	**9.3.1 Freezing process** Potential hazards: viable **parasites**. For killing **parasites** harmful to human health, the freezing temperature and monitoring of duration of freezing should be combined with good inventory control to ensure sufficient cold treatment. **9.4.1 Mincing fish using mechanical separation process**, Potential defects: **parasites. Candling** is recommended for fish suspected of high infestation with **parasites**
	**10.1 General considerations of hazards and defects for frozen surimi production. Parasites** will not be a hazard as the final product will be cooked or pasteurized. **10.1.2 Myxosporidia is a parasite that is common** in marine groundfish such as Pacific whiting. This organism contains protease enzymes that chemically separate proteins that can ultimately affect the gel strength of surimi even at very low incidence. If species are used that are known to contain this **parasite**, protease inhibitors such as beef plasma protein or egg whites may be needed as additives to attain the necessary gel strength capabilities for kamaboko or crab analogue production.
	**12. Processing of salted and dried salted fish, 12.1** Where appropriate, fresh fish intended for processing salted fish should be checked for visible **parasites**. Freezing, heating or adequate combination of salt content and storage time can be used as treatment procedures for killing living parasites. **12.2 Preparing for salting. 12.2.1 Splitting, washing and rinsing**, visible **parasites** should be removed. **12.4.1 Brining: Potential hazards:** viable **parasites. 12.4.2 Brine injection: Potential hazards:** viable **parasites. 12.4.3 Wet-salting: Potential hazards:** viable **parasites. 12.4.4 Dry-salting Potential hazards:** viable **parasites. 12.4.5 Pickling: Potential hazards:** viable **parasites. 12.4.6 Maturing: Potential hazards:** viable **parasites**.
	**13. Smoked fish, smoke-flavoured fish and smoke-dried fish. 13.1 Processing of Smoked Fish:** If raw material likely to contain viable **parasites** is to be used steps must be taken to eliminate this hazard during processing steps, e.g., freezing, heating or salting the product. Alternatively, the final product should be treated in a way to kill **parasites**. **13.1.10 Hot smoking Potential hazards: ****parasites**. **13.1.15 Cooling or freezing Potential hazards: **survival of **parasites**. If freezing at this process step is carried out to kill **parasites**, a time/temperature regime has to be chosen as laid down in Annex I of the Standard for smoked fish, smoke-flavoured fish and smoke-dried fish (CODEX STAN 311-2013). **13.3.2 Smoke-drying. Potential hazards: parasites**.
	**17. Processing of canned fish. 17.3.5.1 Fish preparation: Potential defects: Parasites**.
	**ANNEX I. Potential hazards associated with fresh fish, shellfish and other aquatic invertebrates. 1.1 Parasites** The **parasites** known to cause disease in humans and transmitted by fish are broadly classified as helminths or parasitic worms. These are commonly referred to as **nematodes, cestodes** and **trematodes**. Fish can be parasitized by **protozoans**, but there are no records of fish protozoan disease being transmitted to human beings. Parasites have complex life cycles involving one or more intermediate hosts and are generally passed to human beings through the consumption of raw, minimally processed or inadequately cooked products that contain the parasite infectious stage, causing foodborne disease. Freezing at −20 °C or below for seven days or −35 °C for about 20 h for fish intended for raw consumption will kill **parasites**. Processes such as brining or pickling **may** reduce the parasite hazard if the products are kept in the brine for a sufficient time but may not eliminate it. **Candling, trimming belly flaps** and physically removing the **parasite** cysts will also **reduce the hazards but may not eliminate them**.
	**Nematodes** Many species of nematode are known to occur worldwide and some species of marine fish act as secondary hosts. Among the nematodes of greatest concern are ***Anisakis* spp., *Capillaria* spp., *Gnathostoma* spp**. and ***Pseudoteranova* spp**., which can be found in the liver, belly cavity and flesh of marine fish. An example of a nematode causing disease in human beings is ***Anisakis simplex***; the infective stage of the parasite is killed by heating (60 °C for one minute) and by freezing (−20 °C for 24 h) of the fish core
	**Cestodes** are tapeworms and the species of greatest concern associated with the consumption of fish is ***Dibothriocephalus latus***. This parasite occurs worldwide and both fresh and marine fish are intermediate hosts. Similar to other parasitic infections, the foodborne disease occurs through the consumption of raw or under-processed fish. Similar freezing and cooking temperatures as applied to nematodes will kill the infective stages of this parasite.
	**Trematodes** Fish-borne trematode (flatworm) infections are a major public health problem endemic to approximately 20 countries around the world. The most significant species in terms of the number of people infected belong to the genera ***Clonorchis* and *Ophisthorchis*** (liver flukes), ***Paragonimus*** (lung flukes), and, to a lesser extent, ***Heterophyes*** and ***Echinochasmus (intestinal flukes).*** The most important definitive hosts of these trematodes are human beings or other mammals. Freshwater fish are the second intermediate host in the life cycles of ***Clonorchis*** and ***Ophistorchis***, and freshwater crustaceans in the case of ***Paragonimius.*** Foodborne infections occur through the consumption of raw, undercooked or otherwise under-processed products containing the infective stages of these parasites. Freezing fish at −20 °C for seven days or at −35 °C for 24 h will kill the infective stages of these parasites
**CAC/GL 48-2004** (Last updated 2004) Model certificate for fish and fishery product	
CAC/GL 31-1999 (Last updated 1999) Guidelines for the Sensory Evaluation of Fish and Shellfish in Laboratories	Annexe 1. Belly cavity guts (in intact fish): intact, digested cleanliness (in gutted fish): completely gutted and cleaned, incompletely gutted, not washed belly walls: bright, clean, discoloured, digested **parasites**: absent, present blood: bright, red, brown
**CAC/GL 83-2013** (Last updated 2015) Principles for the use of sampling and testing in international food trade	
**CAC/GL 88-2016** (Last updated 2016) Guidelines on the Application of General Principles of Food Hygiene to the Control of Foodborne Parasites	
**Codex Standard Codes**	**Codex Standard Guideline Which Apply to for Parasite in Seafood Product**
**CODEX STAN 3-1981** (Last updated 2011) Standard for Canned Salmon	
CODEX STAN 36-1981 (Last updated 2017) Standard for Quick Frozen Finfish, Uneviscerated and Eviscerated	**8.4.2 Flesh abnormalities** A sample unit affected by excessive gelatinous condition of the flesh together with greater than 86% moisture found in any individual fish or sample unit with **pasty texture resulting from parasitic** infestation affecting more than 5% of the sample unit by weight.
**CODEX STAN 37-1991 (Last updated 2016)** Standard for Canned Shrimps or Prawns	
**CODEX STAN 70-1981 (Last updated 2016)** Standard for Canned Tuna and Bonito	
**CODEX STAN 90-1981 (Last updated 2016)** Standard for Canned Crab Meat	
**CODEX STAN 92-1981 (Last updated 2017)** Standard for Quick Frozen Shrimps or Prawns	
**CODEX STAN 94-1981 (Last updated 2016)** Standard for Canned Sardines and Sardine-Type Products	
**CODEX STAN 95-1981 (Last updated 2017)** Standard for Quick Frozen Lobsters	
**CODEX STAN 119-1981 (Last updated 2016)** Standard for Canned Finfish	
**CODEX STAN 165-1989 (Last updated 2017)** Standard for Quick Frozen Blocks of Fish Fillets, Minced Fish Flesh and Mixtures of Fillets and Minced Fish Flesh **Amended (2017)**	**7.4 Procedure for the Detection of Parasites** for skinless blocks of fish fillets (Type I method) The entire sample unit is examined non-destructively by placing appropriate portions of the thawed sample unit on a 5-mm thick acryl sheet with 45% translucency and **candled** with a light source giving 1500 lux 30 cm above the sheet.
	**8.3 Parasites** The presence of two or more parasites per kg of the sample unit detected by a method described in 7.4 with a capsular diameter greater than 3 mm or a parasite not encapsulated and greater than 10 mm in length.
**CODEX STAN 166-1989 (Last updated 2017)** Standard for Quick Frozen Fish Sticks (Fish Fingers), Fish Portions and Fish Fillets—Breaded or in Batter	
**CODEX STAN 167-1989 (Last updated 2016)** Standard for Salted Fish and Dried Salted Fish of the Gadidae Family of Fishes	
**CODEX STAN 189-1993 (Last updated 1993)** Standard for Dried Shark Fins	
**CODEX STAN 190-1995 (Last updated 2017)** Standard for Quick Frozen Fish Fillets	**7.4 Procedure for the Detection of Parasites (Type 1 Method) in skinless fillets** The entire sample unit is examined non-destructively by placing appropriate portions of the thawed sample unit on a 5 mm thick acryl sheet with 45% translucency and **candled** with a light source giving 1500 lux 30 cm above the sheet.
	**8.3 Parasites** The presence of two or more parasites per kg of the sample unit detected by the method described in 7.4 with a capsular diameter greater than 3 mm or a parasite not encapsulated and greater than 10 mm in length.
	**8.6 Flesh abnormalities** A sample unit affected by excessive gelatinous condition of the flesh together with greater than 86% moisture found in any individual fillet or a sample unit with pasty texture resulting from **parasitic** infestation affecting more than 5% of the sample unit by weight.
**CODEX STAN 191-1995 (Last updated 1995)** Standard for Quick Frozen Raw Squid	
**CODEX STAN 236-2003 (2003)** Standard for Boiled Dried Salted Anchovies	
**CODEX STAN 244-2004 (Last updated, 2018)** Standard for Salted Atlantic Herring and Salted Sprat	**3.1** Fish Salted Atlantic herring and salted sprats shall be prepared from sound and wholesome fish which are of a quality fit to be sold fresh for human consumption after appropriate preparation. Fish flesh shall not be obviously infested by **parasites**.
	**5.4 Parasites** Fish flesh shall not contain living larvae of nematodes. Viability of nematodes shall be examined according to Annex I. If living nematodes are confirmed, products must not be placed on the market for human consumption before they are treated in conformity with the methods laid down in Annex II
	**7.1 Sampling plan for containers (Barrels)** Sampling of lots for pathogenic microorganisms and parasites will be in accordance with the Principles and Guidelines for the Establishment and Application of Microbiological Criteria Related Foods (CXG 21-1997).
	**8.1.2 Parasites** The presence of readily visible parasites in a sample of the edible portion of the sample unit detected by normal visual inspection of the fish flesh (see Annex III). **ANNEX I VIABILITY TEST FOR NEMATODES** Principle Nematodes are isolated from fish fillets by digestion, transferred into 0.5% Pepsin digestion solution and inspected visually for viability. Digestion conditions correspond to conditions found in the digestive tracts of mammals and guarantee the survival of nematodes. Equipment—Stacked sieves (diameter: 14 cm or larger, mesh size: 0.5 mm)—Magnetic stirrer with thermostated heating plate—Normal laboratory equipment Chemicals—Pepsin 2000 FIP-U/g—Hydrochloric acid Solution A: 0.5% (*w/v*) Pepsin in 0.063 M HCl Procedure Fillets of approximately 200 g are manually shredded and placed in a 2 l beaker containing 1 l Pepsin solution A. The mixture is heated on a magnet stirrer to 37 °C for 1–2 h under continuous slow stirring. If the flesh is not dissolved, the solution is poured through a sieve, washed with water and the remaining flesh is quantitatively replaced in the beaker. 700 mL digestion solution A is added and the mixture stirred again under gentle heating (max. 37 °C) until there are no large pieces of flesh left. The digestion solution is decanted through a sieve and the content of the sieve rinsed with water. Nematodes are carefully transferred by means of small forceps into Petri dishes containing fresh Pepsin solution A. The dishes are placed on a candling dish, and care has to be taken not to exceed 37 °C. Viable nematodes show visible movements or spontaneous reactions when gently probed with dissecting needles. A single relaxation of coiled nematodes, which sometimes occurs, is not a clear sign of viability. Nematodes must show spontaneous movement. Attention When checking for viable nematodes in salted or sugar salted products, reanimation time of nematodes can last up to two hours and more. Remarks Several other methods exist for the determination of viability of nematodes (e.g., ref. 2, 3). The described method has been chosen because it is easy to perform and combines isolation of nematodes and viability test within one step. **ANNEX II Treatment procedures sufficient to kill living nematodes**—e.g., freezing to −20 °C for not less than 24 h in all parts of the product—the adequate combination of salt content and storage time (To be elaborated)—or by other processes with the equivalent effect (To be elaborated) **ANNEX III Determination of the presence of visible parasites** 1. The presence of readily visible parasites in a sample unit that is broken into normal bite-size pieces 20–30 mm of flesh by the thickness of the fillet. Only the normal edible portion is considered even if other material is included with the fillet. Examination should be done in an adequately lighted room (where a newspaper may be read easily), without magnification, for evidence of parasites. 2. Notwithstanding paragraph 1, the verification of the presence of parasites in intermediate entire fishery products in bulk intended for further processing could be carried out at a later stage.
**CODEX STAN 291-2010 (Last updated 2018)** Standard for Sturgeon Caviar	
**CODEX STAN 292-2008 (2015)** Standard for Live and Raw Bivalve Molluscs	
**CODEX STAN 302-2011 (Last updated 2018)** Standard for Fish Sauce	
**CODEX STAN 311-2013 (Last updated 2018)** Standard for Smoked Fish, Smoke-Flavoured Fish and Smoke-Dried Fish	**2.1.2 “Hot smoking” **is a process in which fish is smoked at an appropriate combination of temperature and time sufficient to cause the complete coagulation of the proteins in the fish flesh. Hot smoking is generally sufficient to **kill parasites**, to destroy non-sporulated bacterial pathogens and to injure spores of human health concern.
	**6.3 Parasites** Products covered by this Standard shall not contain living **parasites** and particular attention needs to be paid to cold smoked or smoke-flavoured products, which should be frozen before or after smoking if a **parasite** hazard is present (see Annex 1). Viability of nematodes, cestodes and trematodes shall be examined according to Section 8.10 and/or 8.11.
	**8.10 Determination of the viability of parasites** Methods used for extracting and testing the viability of parasites could include the method set out in Annex I for nematodes in the Standard for Salted Atlantic Herring and Salted Sprat (CXS 244-2004) or other validated methods for parasites acceptable to the competent authority having jurisdiction.
	**8.11 Determination of visible parasites** The entire sample unit is examined for the presence of parasites non-destructively by placing appropriate portions of the thawed (if necessary) sample unit on a 5 mm thick acryl sheet with 45% translucency and candled with a light source giving 1500 lux 30 cm above the sheet.
	**9.2 Parasites** The presence of two or more visible parasites per kg of the sample unit detected by the method described in 8.11 with a capsular diameter greater than 3 mm or a parasite not encapsulated and greater than 10 mm in length.
	**ANNEX I** **Procedures sufficient to kill parasites** A method that is acceptable to the competent authority having jurisdiction shall be used to kill parasites. Where freezing is required to kill parasites (i.e., cold smoked fish and smoke-flavoured fish) the fish must be frozen either before or after processing to a temperature time combination sufficient to kill the living parasites. Examples of freezing processes that may be sufficient to kill some or all parasites are: Freezing at −20 °C at the thermal centre of the product for 24 h (for Anisakis species and Pseudoterranova decipiens only) 1; Freezing at −35 °C at the thermal centre of the product for 15 h (all parasites) 2–5; Freezing at −20 °C at the thermal centre of the product for 168 h (7 days) 2–5 (all parasites).
**CODEX STAN 312-2013 (Last updated 2016)** Standard for Live Abalone and for Raw Fresh Chilled or Frozen Abalone for Direct Consumption or for further Processing	
**CODEX STAN 315-2014 (Last updated 2017)** Standard for Fresh and Quick Frozen Raw Scallop Products (2014)	**8.6 Examination for Parasites** The presence of readily visible parasites in a sample unit detected by normal visual inspection of the scallops.
	**9.4 Parasites** The presence of parasites should not be at an objectionable level.
**CODEX STAN 329-2017 (Last updated 2017)** Standard for Fish Oils	
